# NHC-catalyzed covalent activation of heteroatoms for enantioselective reactions

**DOI:** 10.1039/d1sc00469g

**Published:** 2021-03-02

**Authors:** Runjiang Song, Zhichao Jin, Yonggui Robin Chi

**Affiliations:** Laboratory Breeding Base of Green Pesticide and Agricultural Bioengineering, Key Laboratory of Green Pesticide, Agricultural Bioengineering Ministry of Education, Guizhou University Huaxi District Guiyang 550025 China; Division of Chemistry & Biological Chemistry, School of Physical & Mathematical Sciences, Nanyang Technological University Singapore 637371 Singapore robinchi@ntu.edu.sg

## Abstract

Covalent activation of heteroatoms enabled by N-heterocyclic carbene (NHC) organic catalysts for enantioselective reactions is evaluated and summarized in this review. To date, sulfur, oxygen, and nitrogen atoms can be activated in this manner to react with another substrate to construct chiral carbon–heteroatom bonds with high optical enantioselectivities. The activation starts with addition of an NHC catalyst to the carbonyl moiety (aldehyde or imine) of substrates that contain heteroatoms. The key in this approach is the formation of intermediates covalently bound to the NHC catalyst, in which the heteroatom of the substrate is activated as a nucleophilic reactive site.

## Abstract 2

Covalent activation of heteroatoms enabled by N-heterocyclic carbene (NHC) organic catalysts for enantioselective reactions is evaluated and summarized in this review.

## Graphical abstract

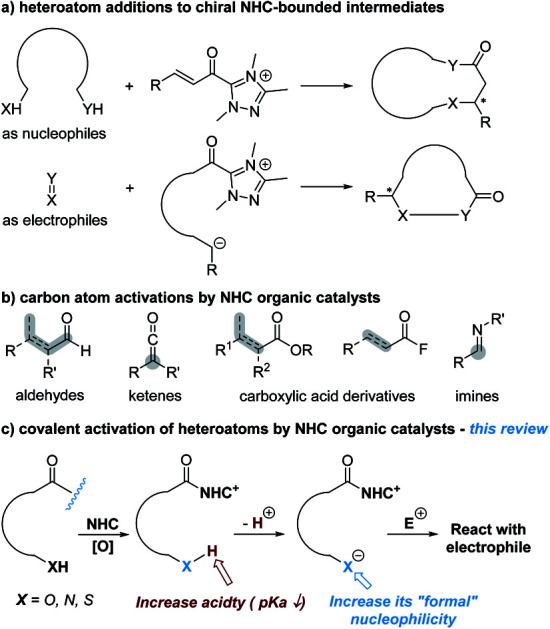

## 1. Introduction

**Fig. 1:**
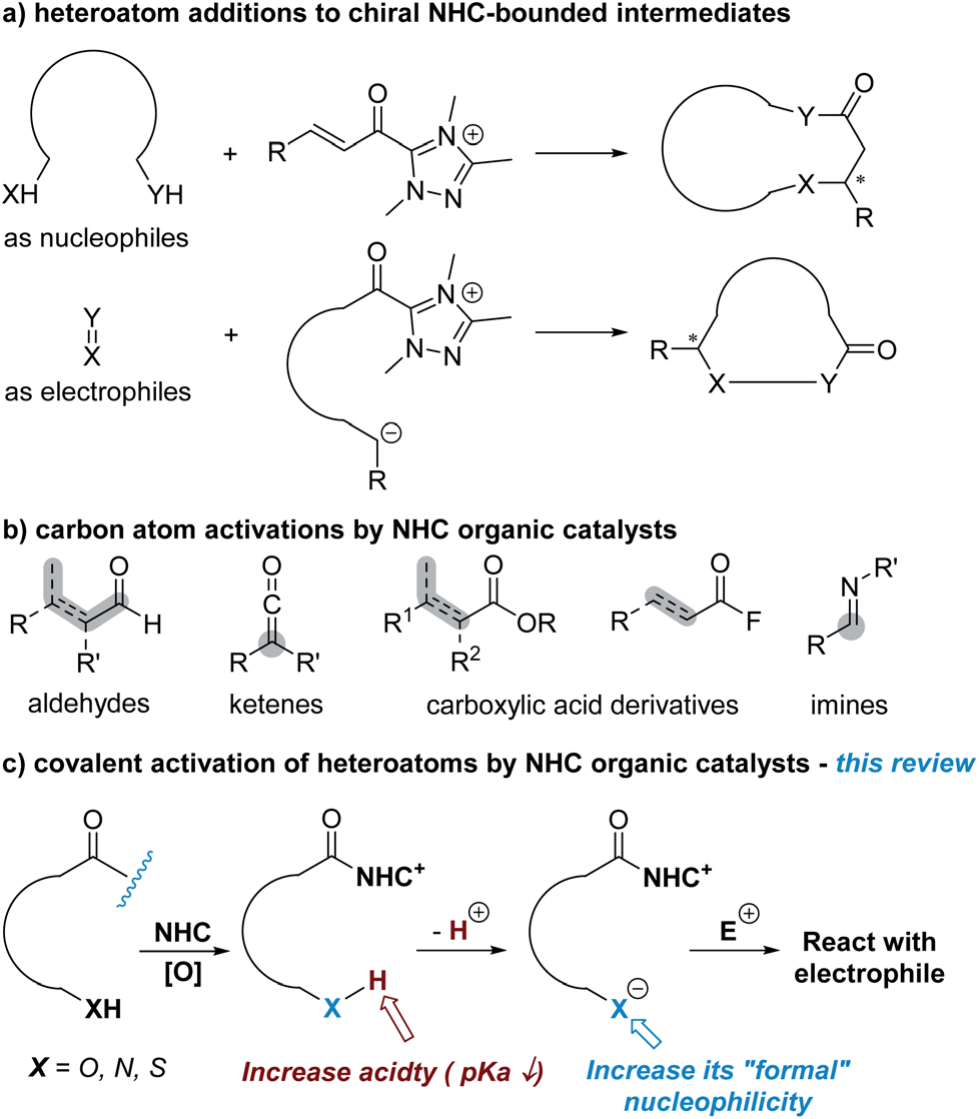
NHC-catalyzed activation of carbon and heteroatoms.

Heteroatoms are essential to life of all kinds and ubiquitous in nearly all functional molecules with medicinal uses. Asymmetric construction of carbon–heteroatom (C–X) bonds has therefore been an active research topic in chemical synthesis. In the last two decades, catalysis enabled by N-heterocyclic carbenes (NHCs) has been developed as a versatile approach to prepare both carbon–carbon and carbon–heteroatom bonds in highly enantioselective manners.^1^ In the subdomain of NHC-catalyzed asymmetric C–X bond formation, the dominant approach is to activate carbon atoms to react with heteroatoms or substrates containing heteroatoms such as amines, ureas and thioureas (Fig. 1a). The carbon atoms can be activated as electrophilic reaction centres *via* NHC-derived (unsaturated) acyl azolium intermediates or their analogues to react with nucleophilic heteroatoms.^2^ Alternatively, the carbon atoms are activated as nucleophilic reaction centers (*via* acyl anion and enolate intermediate or their analogues). The NHC-derived nucleophilic intermediates then react with heteroatoms (in their oxidized states) or heteroatom-containing substrates (such as imines) as the electrophiles.^3^Fig. 1b shows representative substrates with carbon atoms activated by NHC catalysts as the reactive centres. The heteroatoms involved in these reactions, at least by designing, are not activated by NHC catalysts. These types of reactions have now been covered in many review articles concerning NHC catalysis from different perspectives.

A much less-developed approach is to activate heteroatoms through addition of NHC catalysts to the respective substrates for enantioselective reactions. The basic design, as illustrated in Fig. 1c, is to increase the reactivity of heteroatoms by forming covalent bonds between NHC catalysts and substrates. This review is to summarize the development of this direction that is still at its infant stage.

It is worth noting that activation of heteroatoms for asymmetric reactions *via* non-covalent interactions using azolium salts (precursor of N-heterocyclic carbene), pioneered by Huang and co-workers, is not covered in this article.^4^ It is also important to note that in those reactions designed *via* NHC-catalyzed activation of carbon atoms as the key step, heteroatoms in the substrates or intermediates may also be activated as a result of catalyst–substrate bonding. These studies are also not discussed in this article.

With the scope defined, this review article will discuss NHC-catalyzed activation of oxygen and nitrogen atoms for enantioselective reactions. The use of NHC catalysts to cleave the N–S or C–S bond to release nucleophilic sulfur atoms for asymmetric reactions will also be described.

## 2. Covalent activation of oxygen atoms of phenols

Aromatic aldehydes are routinely activated by NHC catalysts and participate in Benzoin condensations, Stetter reactions and acylation reactions. In 2013, the Chi lab found that the benzylic C(sp^3^)–H bond of an indole aldehyde can be activated by NHC catalysts under oxidative conditions through the formation of an *o*-QDM intermediate.^5^ This study suggests that the activating power of NHC catalysts can go across the conjugated aromatic ring system.

**Fig. 2:**
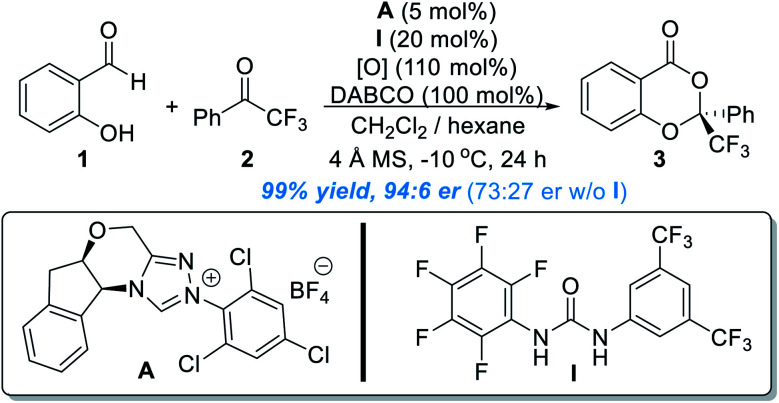
Covalent activation of phenol OH by chiral NHC.

Encouraged by this discovery, the Chi group moved to explore the activation of heteroatoms attached to aromatic scaffolds. In 2017, they disclosed that phenol OH can be enantioselectively functionalized through activation of salicylaldehyde by NHC organic catalysts (Fig. 2).^6^ In a model reaction using salicylaldehyde (**1**) and trifluoroacetophenone (**2**) as substrates, the use of **A** as the NHC pre-catalyst (5 mol%) in the presence of **I** as an additive (20 mol%) and an oxidant gave the chiral acetal product (**3**) in 99% yield, 94 : 6 er. The urea additive was critical and behaved as a co-catalyst. The product er dropped to 73 : 27 in the absence of the urea additive. The reaction appears to be general with good functional group tolerance, as shown in 28 examples. The NHC catalyst loading can be decreased in 1 mol% for gram scale synthesis. The authors also found that several of their enantiomerically enriched products showed encouraging antifungal activities against Eggplant Verticillium, Phytophthora infestans and Fusarium oxysporum.

**Fig. 3:**
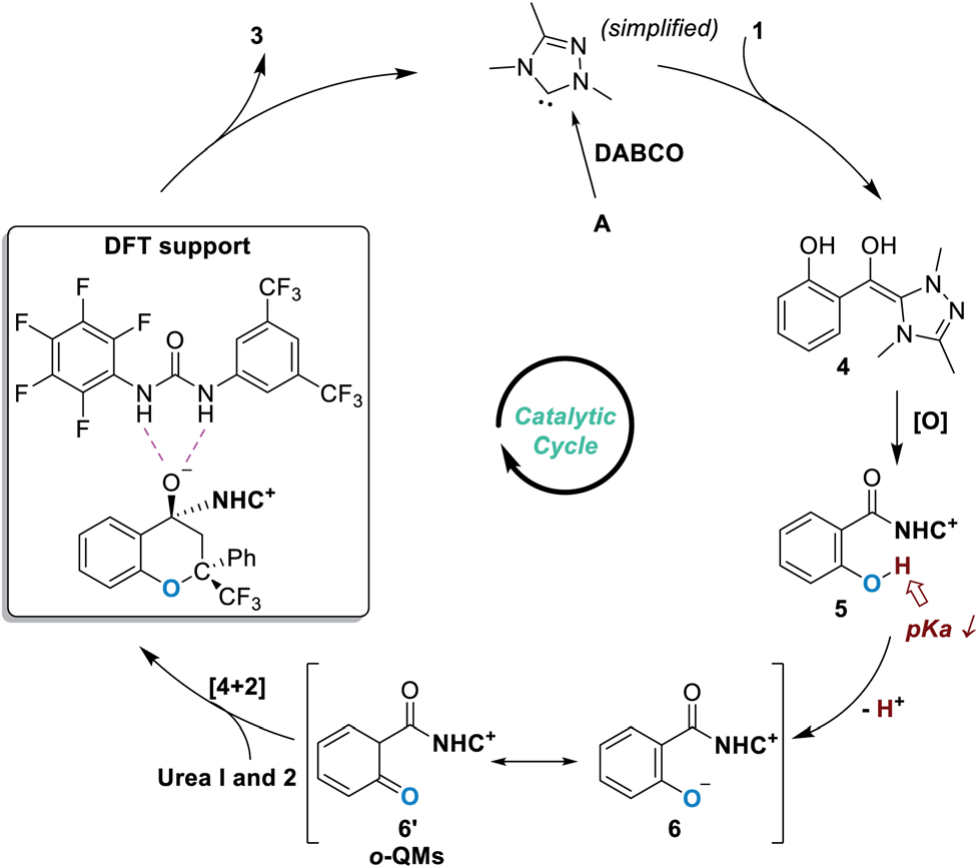
Proposed mechanism of the NHC-catalyzed phenol OH activation reaction.

Based on experimental observations and DFT calculations, the reaction was believed to proceed through a pathway as illustrated in Fig. 3. Addition of the NHC catalyst to salicylaldehyde **1** gives the Breslow intermediate **4**, which can be oxidized to produce the acylazolium intermediate **5**. Deprotonation of **5** under basic conditions leads to the formation of the zwitter ionic phenoxide **6**, which is stabilized through resonance structures including the *o*-QMs **6′**. The asymmetric oxa-[4 + 2] reaction between **6** (**6′**) and **2** gives the chiral acetal product **3** and releases the free NHC catalyst for additional catalytic cycles.

## 3. Covalent activation of nitrogen atoms

**Fig. 4:**
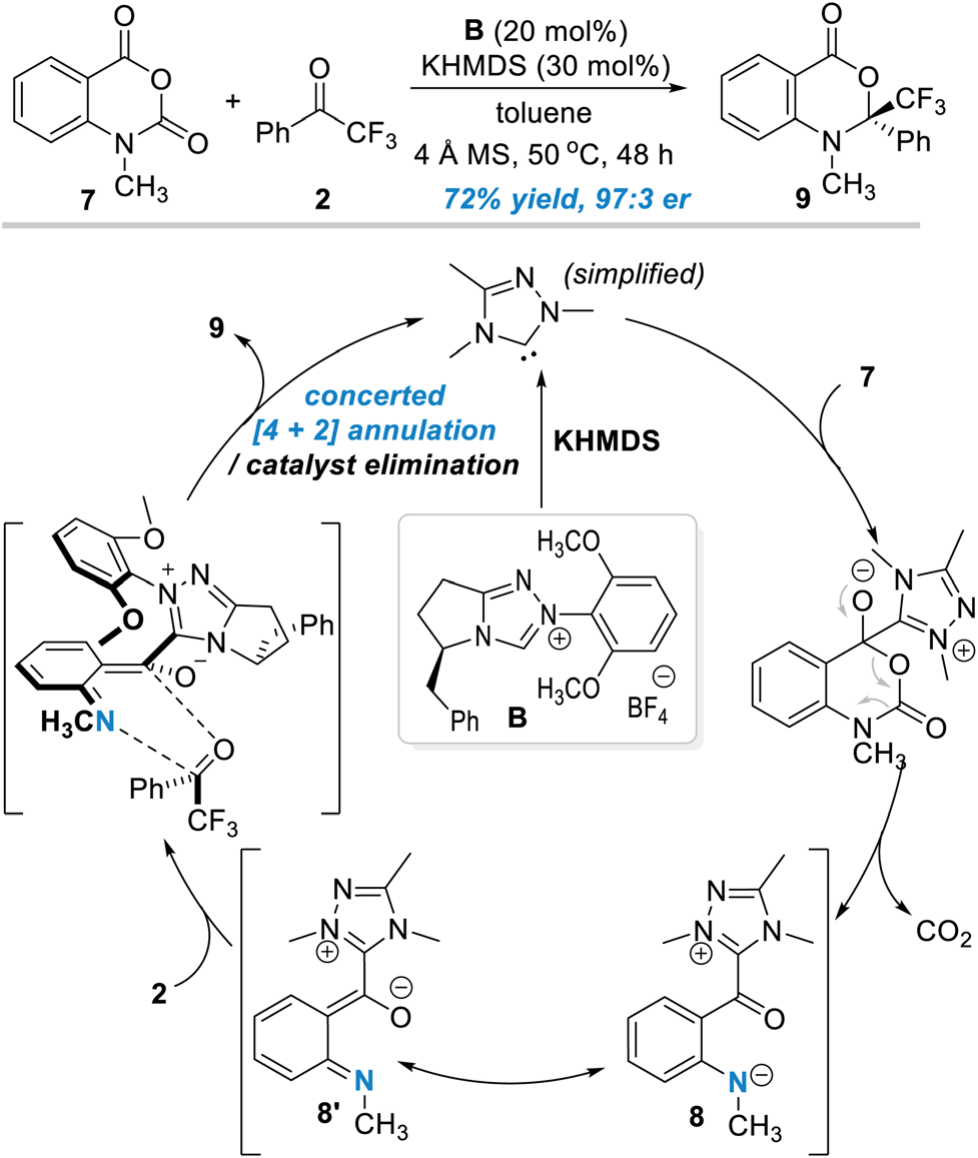
Covalent activation of the nitrogen atom of *N*-methylisatoic anhydride by the chiral NHC catalyst.

The oxygen atom in phenols above can in principle be replaced by other heteroatoms such as nitrogen and sulphur atoms for NHC-catalyzed enantioselective reactions. An elegant design in this direction was disclosed by Scheidt and co-workers in 2019 (Fig. 4).^7^ Addition of a carbene catalyst (**B**) to an *N*-methylisatoic anhydride (**7**) gives zwitter ionic acylazolium intermediate **8** with elimination of one equivalent of CO_2_. Intermediate **8** is a resonance structure of the *aza-o*-QMs intermediate **8′**. DFT studies suggest the intermediate **8**/**8′** reacts with trifluoroacetophenone **2** through a concerted enantioselective [4 + 2] annulation pathway. The target product (**9**) is obtained with an excellent enantioselectivity. Common functional groups are well tolerated in this reaction, and the authors demonstrated 19 examples in their substrate scope evaluation.

**Fig. 5:**
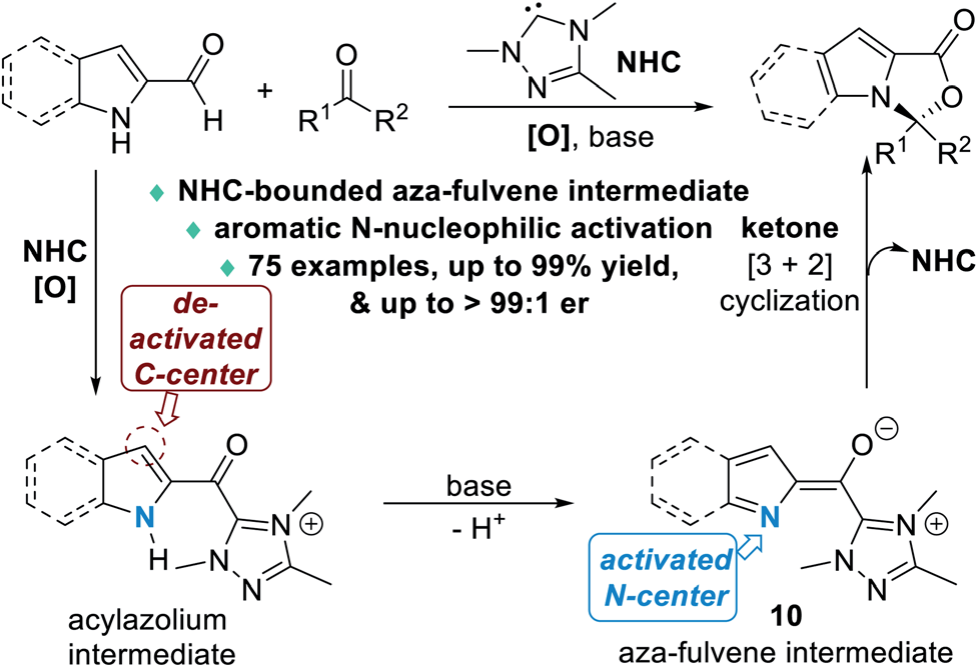
NHC-catalyzed nucleophilic activation of aromatic N atoms of indole-2-carbaldehydes or pyrrole-2-carbaladehydes.

Nitrogen atoms that are part of the aromatic scaffold can also be activated by NHC catalysts for enantioselective reactions. In 2020, Chi and Jin disclosed that the aromatic N atoms of the 2-carbaldehyde derivatives of indole or pyrrole structures can be effectively activated *via* the formation of an unprecedented NHC-bounded aza-fulvene intermediate **10** (Fig. 5).^8^ The N atoms of the indole or pyrrole molecules are selectively activated to react with various ketone substrates through nucleophilic addition reactions.

**Fig. 6:**
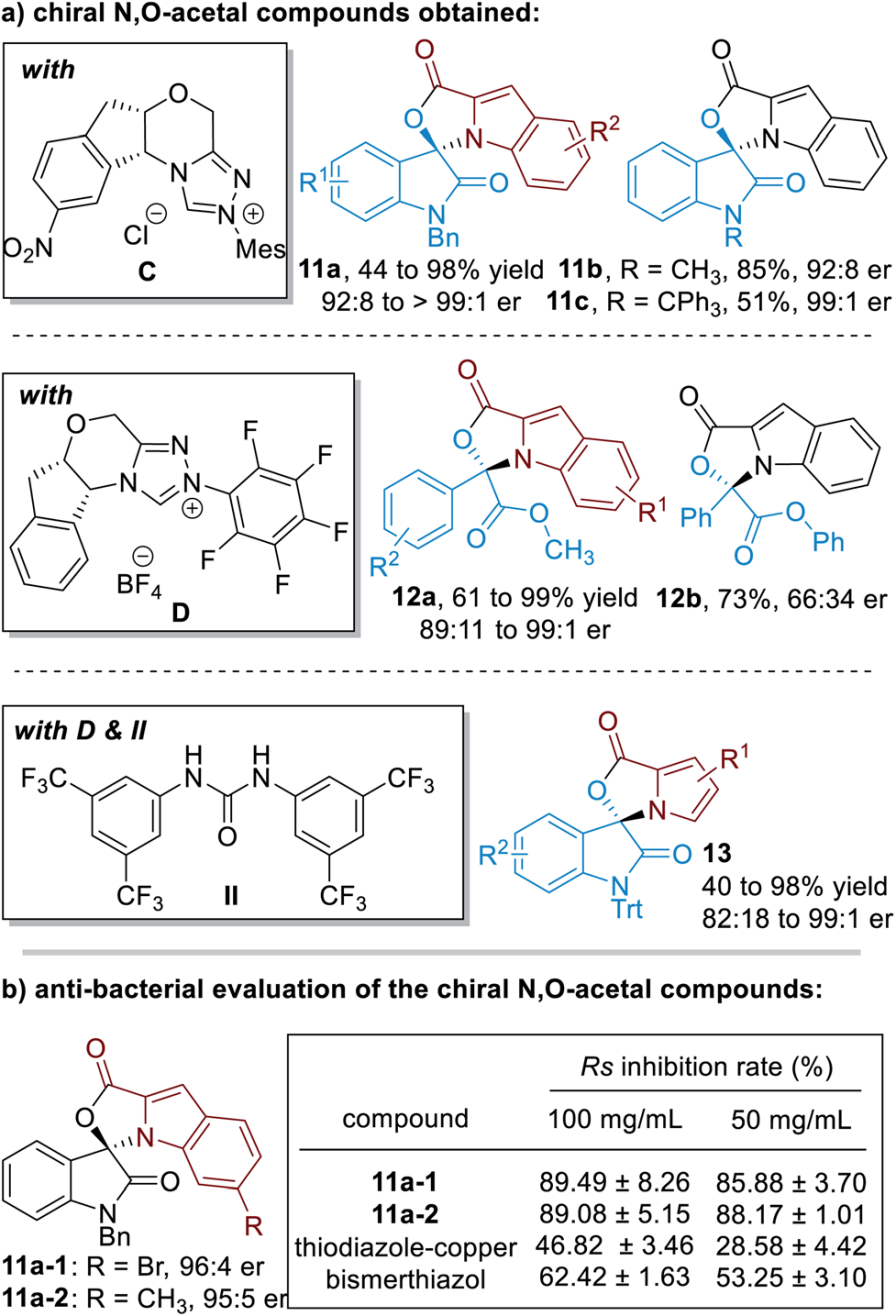
Chiral N,O-acetals prepared through NHC organocatalytic reactions and their bioactive evaluations.

Up to 75 enantiomerically enriched N,O-acetal compounds have been efficiently prepared through this protocol under the catalysis of several chiral NHC organic catalysts (Fig. 6a). For example, with chiral NHC **C** used as the reaction catalyst, spiro-cyclic compounds **11** can be obtained in moderate to excellent yields with excellent optical purities. Switching the NHC catalyst to **D**, α-ketoesters can be applied as suitable electrophiles to react with substituted indole-2-carbaldehydes (**12**). Pyrrole-2-carbaldehydes bearing different substituents can also work well in this process, although a urea co-catalyst **II** is needed for better enantioselectivity control over the NHC-catalyzed [3 + 2] cycloaddition reaction (**13**).

It is worth noting that several of these spiro-cyclic compounds have shown promising anti-microbial activities (Fig. 6b). For instance, compounds **11a-1** and **11a-2** exhibit much better anti-bacterial activities against *Ralstonia solanacearum* (abbreviated as *Rs*) than the commercial agrichemicals thiodiazole–copper and bismerthiazol.

A short time later, Biju,^9^ Hui^10^ and co-workers independently reported similar transformations to this protocol. DFT studies towards the NHC-catalyzed aromatic N activation mechanism and additional electrophile investigations are provided in their studies.

**Fig. 7:**
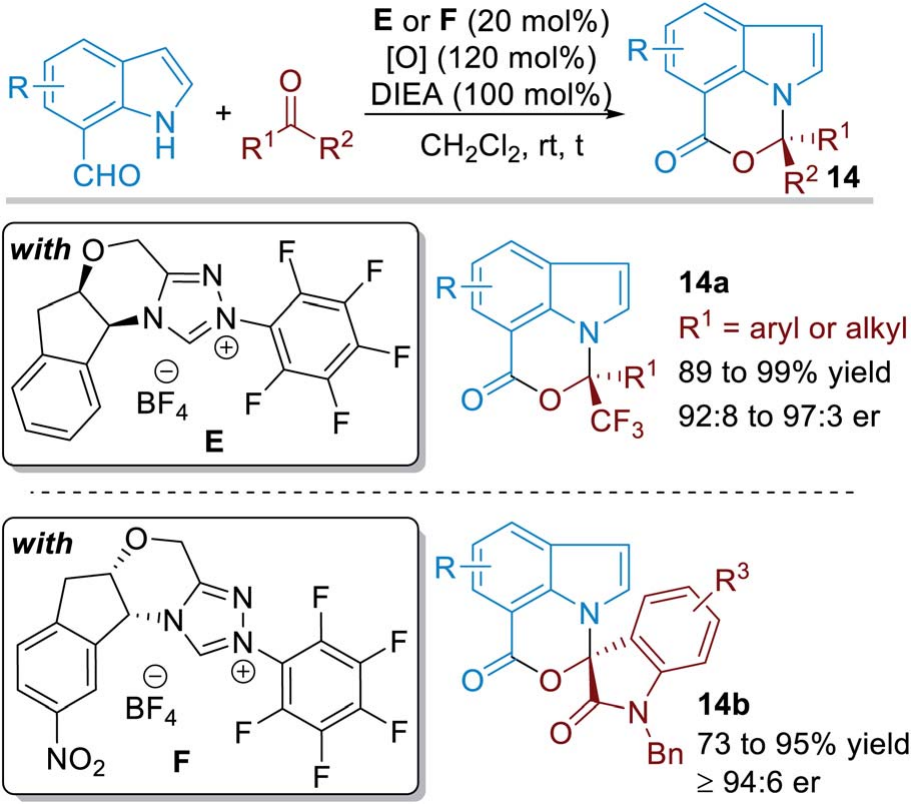
NHC-catalyzed enantioselective activation of indole-7-carbaldehydes.

When the aldehyde group was installed on the 7-position of the indole structure, the aromatic N atom can also be functionalized through covalent activations with NHC organic catalysts (Fig. 7).^11^ In this case, various electron-deficient ketone substrates can be used as electrophiles. With the catalysis of NHC **E** or **F**, a variety of chiral [1,3]oxazino[5,4,3-*hi*]indole derivatives **14** can be afforded in good to excellent yields and enantioselectivities.

**Fig. 8:**
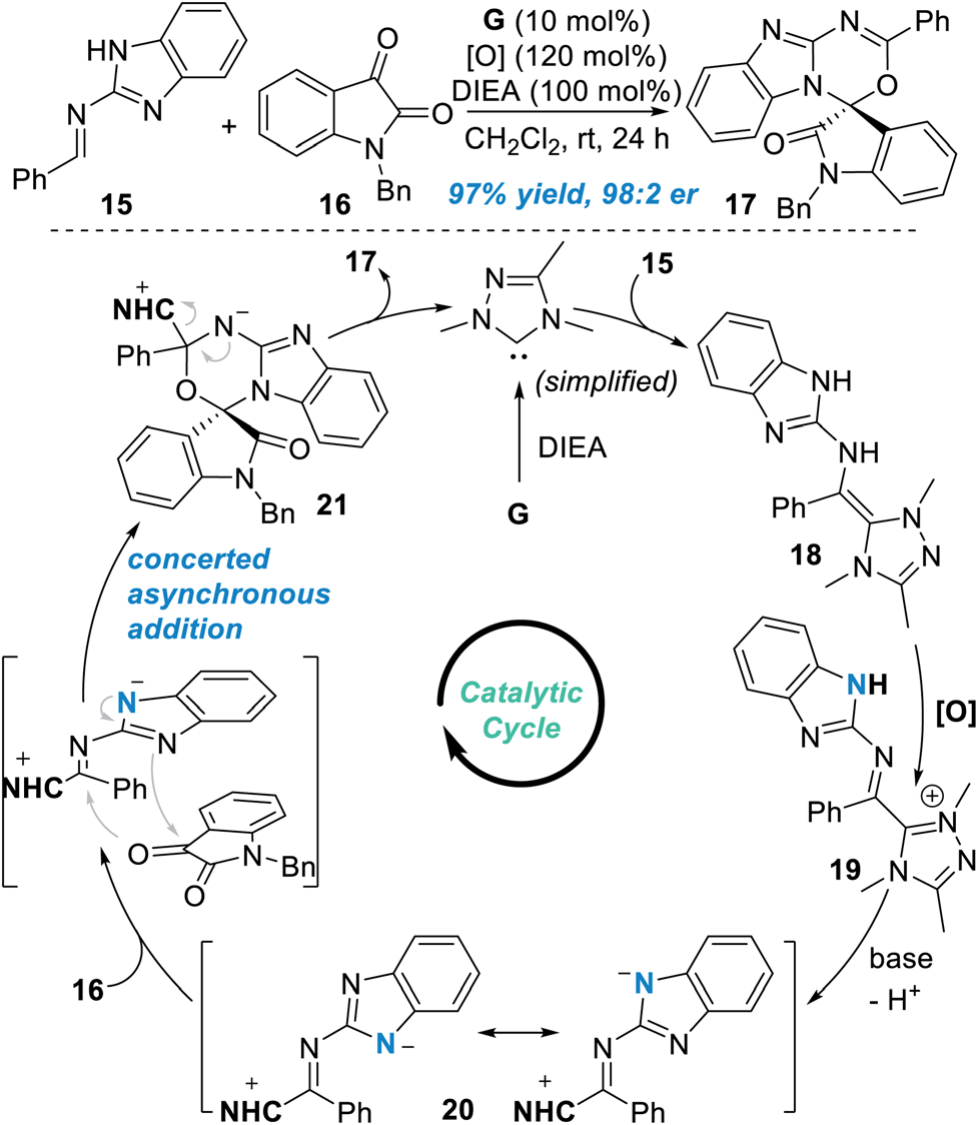
NHC-catalyzed enantioselective activation of the guanidine-derived aldimine.

Very recently, the groups of Chi^12^ and Fu^13^ independently found that the remote N atom of the guanidine-derived aldimine **15** can be activated by NHC catalyst **G** under oxidative conditions to react with isatin **16** and gave spiocyclic product **17** in an excellent yield and enantioselectivity (Fig. 8). Addition of the NHC catalyst **G** to **15** leads to the formation of the *aza*-Breslow intermediate **18**, which can be transformed to give the azolium intermediate **19** under oxidative conditions. Deprotonation of **19** gives the triaza-diene intermediate **20**, which then reacts with isatin **16** through a concerted asynchronous addition process to give the intermediate **21**. Finally, the target chiral spirocyclic product **17** can be afforded after elimination of the NHC catalyst.

**Fig. 9:**
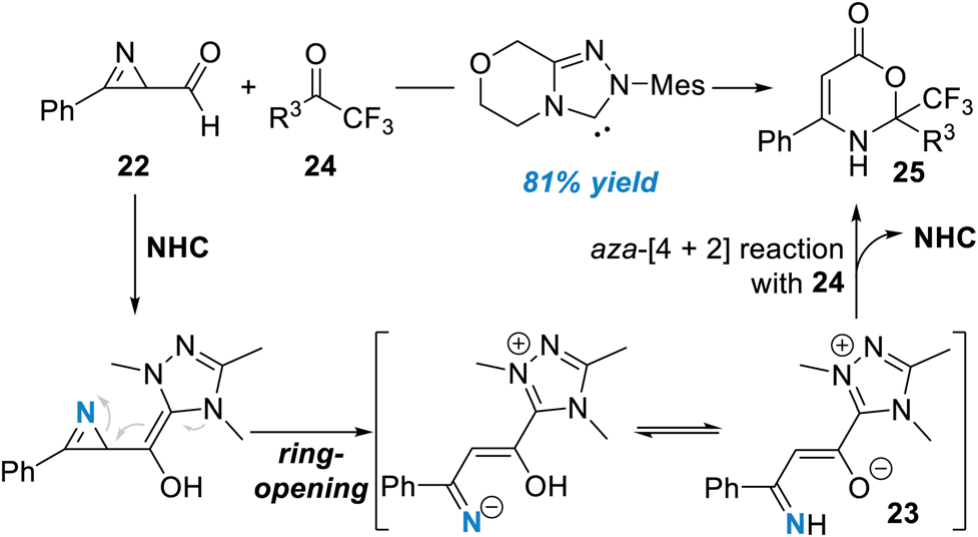
Activation of azirines by the NHC organic catalyst.

Another interesting study in this direction is activation of the nitrogen atoms of azirines (Fig. 9).^14^ In this study, formyl azirine **22** was successfully activated by the NHC catalyst and a novel azolium *aza*-dienolate intermediate **23** was generated. *Aza*-[4 + 2] cycloaddition between **23** and the electron-deficient ketone **24** gave the oxazinone product **25** in moderate to excellent yields. Although a wide range of substrates with various substitution patterns worked well in this process, the asymmetric version of this transformation remains undisclosed.

## 4. NHC-catalyzed C–S and N–S bond cleavage to release sulphur atoms for enantioselective reactions

**Fig. 10:**
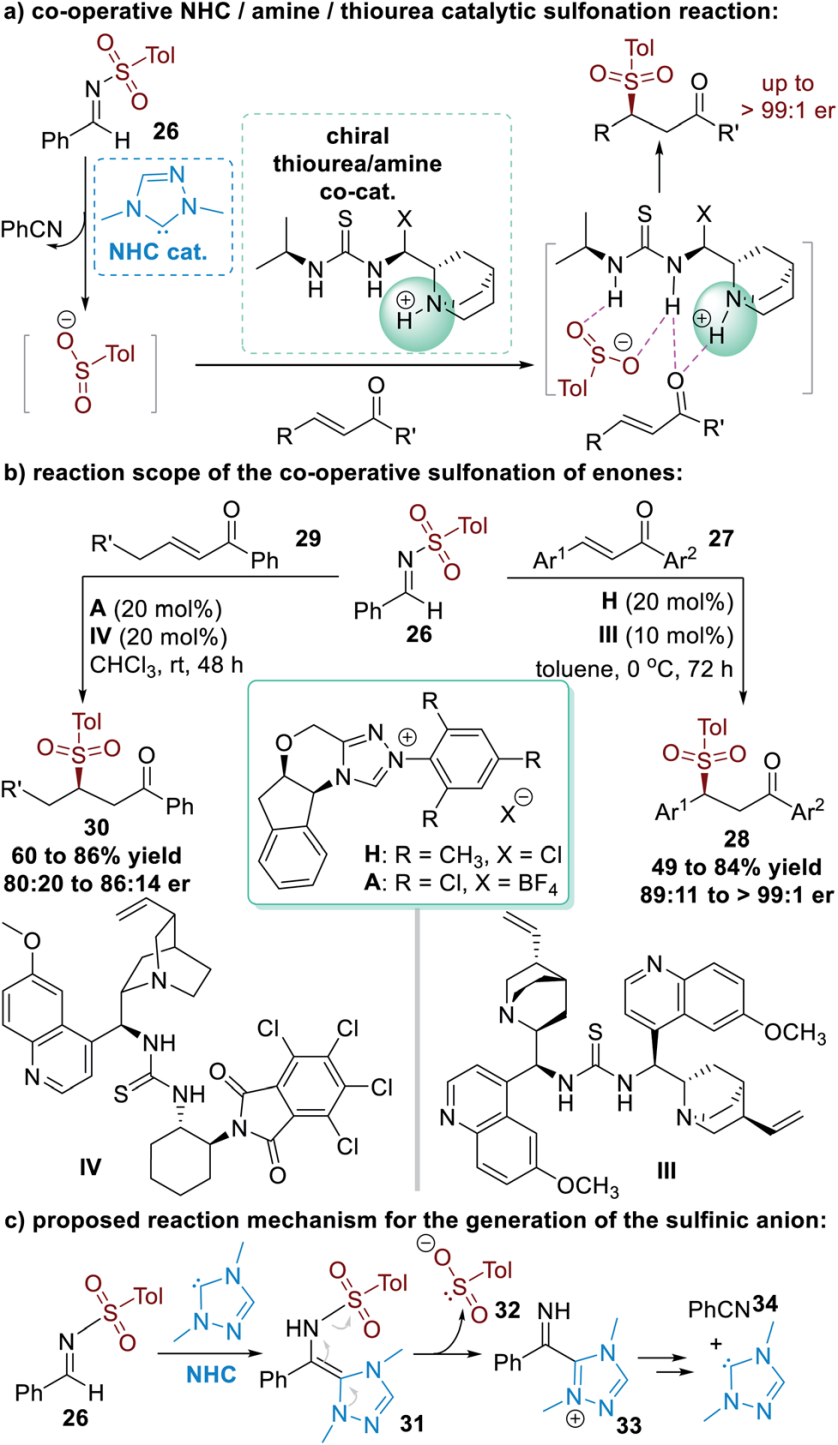
NHC-catalyzed sulfonimide N–S bond cleavage.

The N–S bond in aldehyde-derived sulfonimide (**26**) was cleavable as a result of NHC-catalyzed activation, as disclosed by Hou and co-workers in 2008.^15^ This N–S bond cleavage releases nucleophilic sulfinic anions that can react with other electrophiles. In these reactions, since the NHC catalyst is not bound to the sulfinic anions, enantioselectivity control cannot be induced by chiral NHC catalysts. In 2013, Chi and co-workers introduced a multi-catalytic approach combining NHC and a thiourea/tertiary amine multifunctional catalyst to achieve enantioselective transformations (Fig. 10a).^16^

Aromatic enone substrates **27** worked well in this co-operative catalytic process, with the β-sulfinic ketone **28** afforded in moderate to good yields and good to excellent enantioselectivities (Fig. 10b, right part). NHC pre-catalyst **H** and the chiral amino-thiourea **II** were used as the co-operative catalysts for this asymmetric transformation. β-Alkyl substituted enones **29** were also promising substrates for this reaction, although the yields and er values of the final products **30** were only moderate even after additional condition optimization investigations (Fig. 10b, left part).

The NHC catalyst is believed to first attack the sulfonimide **26** through nucleophilic addition and give the *aza*-Breslow intermediate **31**. The *aza*-Breslow intermediate **31** readily cracks into the sulfinic anion **32** and the azolium cation **33**. Thio-Michael addition reactions of **32** and enones (**27** or **29**) and intermolecular proton transfer lead to the formation of the β-sulfinic ketone products (**28** or **30**) together with the side-product benzonitrile **34**. The free NHC catalyst is regenerated during this process and can participate in further catalytic cycles (Fig. 10c).

The NHC-catalyzed N–S bond cleavage strategy has recently been used in the late-stage functionalization of bio-active complex sulfonamides.^17^ The key intermediate of the sulfonimide can be formed *in situ* from benzaldehyde and the corresponding sulfonamide. Therefore, various sulfonamide substrates can be de-aminated and transformed to other sulfone-containing functional molecules through a one-pot protocol in the presence of benzaldehyde and an NHC organic catalyst.

**Fig. 11:**
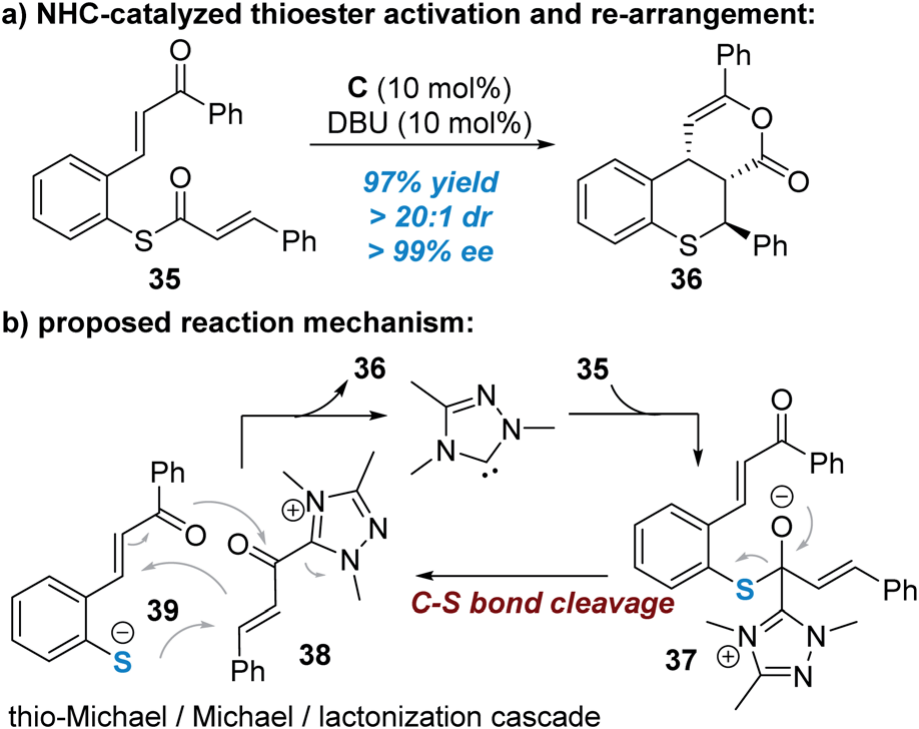
NHC-catalyzed N–S bond cleavage of thioesters.

The C–S bond of thioesters derived from thiophenols can be broken to release thiophenol with the sulphur atom reacting as a nucleophile, as reported by Xu and co-workers (Fig. 11).^18^ In this reaction, the enantioselectivity is controlled by the other reacting partner (α,β-unsaturated acyl azolium ester intermediate) connected with the NHC catalyst.

Nucleophilic attack of the thioester **35** by the NHC catalyst generates the zwitter ion **37**, which can be broken up to give the α,β-unsaturated acylazolium intermediate **38** and the thio anion **39**. A stereoselective cascade thio-Michael addition/Michael addition/lacton formation process between the ionic intermediates **38** and **39** finally affords the chiral product **36** and liberates the free NHC catalyst for additional catalytic cycles (Fig. 11b).

## 5. Summary and outlook

NHC organic catalysis has been proven to be a versatile strategy in asymmetric synthesis. The extraordinary development in the past two decades, on the other hand, has mainly focused on activation of carbon atoms for enantioselective reactions. Activation of heteroatoms by NHCs for asymmetric reactions is less explored. This review highlights the impressive yet still very limited studies in this direction. Specifically, oxygen, nitrogen, and sulphur atoms in relevant substrates have been activated by NHCs as nucleophiles for a number of highly enantioselective reactions. These reactions involve previously unexplored NHC-bound intermediates and present interesting reactivities. It is expected that this new approach will allow for quick construction of functional molecules, especially those bearing multiple heteroatoms, in a much simpler manner that is not achievable with the more matured approach of carbon atom activation. At present, the potential in activating heteroatoms by NHCs does not seem to be realized to any considerable level. We hope that this short review will encourage further development in this arena.

## Conflicts of interest

There are no conflicts to declare.
